# Antibacterial and Antibiofilm Activity of Temporin-GHc and Temporin-GHd Against Cariogenic Bacteria, *Streptococcus mutans*

**DOI:** 10.3389/fmicb.2019.02854

**Published:** 2019-12-11

**Authors:** Hengren Zhong, Zhipeng Xie, Hanqi Wei, Shuxia Zhang, Yanting Song, Manchuriga Wang, Yingxia Zhang

**Affiliations:** ^1^Key Laboratory of Tropical Biological Resources of Ministry of Education, Department of Pharmaceutics, School of Life and Pharmaceutical Sciences, Hainan University, Haikou, China; ^2^Department of Animal Medicine, College of Animal Science, Hainan University, Haikou, China

**Keywords:** antimicrobial peptides, biofilm, *Streptococcus mutans*, extracellular polysaccharides, glucosyltransferases

## Abstract

Temporin-GHc (GHc) and temporin-GHd (GHd) produced by the frog *Hylarana guentheri* had shown broad-spectrum antibacterial activities against bacteria and fungi. In this study, we investigated whether they exert antibacterial and antibiofilm activities against cariogenic bacteria, *Streptococcus mutans*. GHc and GHd adopt the random coil conformation in aqueous solution and convert to α-helix in membrane mimetic environments by using circular dichroism spectroscope. They are positively charged by histidine, with the polar and nonpolar amino acids on opposing faces along the helix. The amphipathicity enabled the peptides to target at bacterial membrane, leading to an increase in membrane permeation and disruption of *S. mutans*, which allowed the peptides to bind with genomic DNA. GHc and GHd completely impeded the initial attachment of biofilm formation and disrupted preformed *S. mutans* biofilms. The expression of exopolysaccharide (EPS) biosynthesis genes which synthesize glucosyltransferases in *S. mutans* was downregulated by exposing to GHc or GHd, contributing to the decrease of soluble and insoluble EPS. GHc and GHd exhibited selectivity toward *S. mutans* in the presence of human erythrocytes, and no cytotoxicity toward human oral epithelial cells was observed at a concentration of 200 μM. These results laid the foundation for the development of GHc and GHd as potential anti-caries agents.

## Introduction

Dental caries, the current most prevalent infectious oral disease, is caused by pathogenic bacteria and affects many children and most adults (Xiao et al., 2012; Koo et al., 2013). *Streptococcus mutans* has been identified as one of the major causing bacteria, which adheres to the tooth surface, grows into colonies, and produces covering biofilms. The bacterial communities of *S. mutans* in biofilms utilize carbohydrates to produce organic acids, which has been attributed to enamel corrosion and dentin damage (Quirynen and Bollen, 1995; Nakano et al., 2009). Glucosyltransferases (GTFs) play critical function in synthesizing both soluble and insoluble extracellular polysaccharides (EPSs) in *S. mutans* (Hanada and Kuramitsu, 1988; Thomas et al., 2013; Hoque et al., 2016). The EPSs help *S. mutans* attach to the tooth surface and promotes formation of biofilms, which are partially responsible for the antibiotic resistance of bacteria (Flemming et al., 2007; Xiao et al., 2012). Thus, inhibiting the production of EPS can hinder the biofilm formation and decrease the occurrence of dental caries.

Over the past decades, widespread antibiotics use and their abuse have brought in various antibiotic-resistant bacteria, which are becoming more and more elusive to conventional antibiotic treatment (Perreten et al., 1997; Tacconelli et al., 2017); this renders it urgent to develop new antibiotic agents (Koczulla and Bals, 2003). Antimicrobial peptides (AMPs), found in microorganisms, plants, and animals, exhibit wide spectrum of activities against microbial pathogens and play an important role in host immune defense (Seo et al., 2012). Usually, positively charged AMPs exert their effects through charge interactions with the anionic membrane lipids of the bacteria, thus making it difficult for them to develop resistance against AMPs (Fjell et al., 2012). AMPs have been considered as the promising novel antibiotic candidates in the fight against bacterial infections. In contrast with other AMPs, the temporin superfamily shares the features of a short, highly conserved N-terminal polypeptide with 13–14 amino acid residues and a low net positive charge (0 to +3) (Michael, 2002; Rollinssmith et al., 2003; Abbassi et al., 2008).

Temporin-GHc (GHc, GenBank accession No: KU518308) and temporin-GHd (GHd, GenBank accession No: KU518309) are 13-residue AMPs cloned from *Hylarana guentheri* with broad-spectrum antimicrobial activities. They showed similar antimicrobial activities against both Gram-positive and Gram-negative bacteria (Dong et al., 2017). In this study, we investigated the antimicrobial activities of GHc and GHd against *S. mutans*, examined their effects on the *S. mutans* biofilm formation and integrity, and assessed their impacts on *S. mutans* virulence factors, such as EPSs production and GTFs expression.

## Materials and Methods

### Bacterial Strain

*S. mutans* (ATCC25175) used in this study was purchased from Guangdong Institute of Microbiology (Guangzhou, China). *S. mutans* was grown in brain heart infusion broth (BHI, Beijing Land Bridge, China) at 37°C under anaerobic conditions in an AnaeroPack rectangular jar (Mitsubishi Gas Chemical Company, Inc., Japan).

### Synthesis of Peptides

GHc and GHd were commercially synthesized (Jier Biotech Co., Ltd., Shanghai, China) through the Fmoc-based solid-phase peptide synthesis protocol. The identities of the obtained peptides were checked by mass spectrometry, and their purities (>95%) were verified by reverse phase high-performance liquid chromatography (RP-HPLC) (Supplementary Figures S1, S2).

### Circular Dichroism Spectroscopy and Predicted Peptide Structure Analysis

The circular dichroism (CD) spectroscopy was performed in a Bright time Chirascan spectropolarimeter (Applied Photophysics Limited, UK). The peptides were dissolved at the final concentration of 150 μM in 50% trifluoroethanol (TFE), 30 mM sodium dodecyl sulfate (SDS) micelles or 10 mM phosphate buffered saline (PBS, pH 7.2). Helical Wheel Projections^1^ were applied to predict the secondary structures of GHc or GHd and PyMOL software was employed to theoretically illustrate their 3-dimentional (3D) structures.

### Antibacterial Activities of GHc and GHd Against *Streptococcus mutans*

#### Determination of the Minimum Inhibitory Concentration and Minimum Bactericidal Concentration

The two-fold dilution method was used to determine the minimum inhibitory concentration (MIC) of the peptides as described previously (Leoni et al., 2017). *S. mutans* in the exponential phase was diluted to 2 × 10^6^ CFU/ml in BHI. Fifty microliters of GHc or GHd were prepared as a two-fold serial dilution with water in 96-well microtiter plates. Then, 50 μl of diluted bacterial suspension (final concentration of 10^6^ CFU/ml) was added to each well of the plates. The plates were incubated at 37°C under anaerobic conditions for 16 h. The absorbance at 600 nm was detected with a microplate reader (Multiskan Spectrum, BioTek Inc., USA). Non-treated bacteria and BHI broth medium were used as a negative control and blank, and chlorhexidine was used as the positive control. The MIC was defined as the lowest concentration of GHc or GHd that inhibited bacterial growth. Minimum bactericidal concentrations (MBCs) were determined by plating the bacterial suspensions from the MIC assay wells at the peptide concentrations equal to or higher than the MIC values on solid agars. The lowest peptide concentrations at which there was no bacterial growth on plates after incubation were taken as the MBC (Liu et al., 2017). Resazurin microtiter assay was also performed as previously described to determine the MIC (Palomino et al., 2002). All sets were conducted in triplicate.

#### Growth Curve Assay of GHc and GHd

To eliminate the interference caused by bacteria numbers between the peptide-treated and untreated treatments, the amounts of bacteria should be kept equal in the extraction of EPS and quantitative real-time PCR (RT-qPCR) assay. So the growth curves of *S. mutans* treated with the peptide concentrations below their respective MIC were performed. *S. mutans* in the exponential phase was diluted in BHI to 2 × 10^6^ CFU/ml. One hundred microliters of GHc or GHd (the final concentration of 0.25×, 0.5× and 1× MIC) were prepared in 96-well microtiter plates. The same volume of bacterial suspension was added in each well. The plates were incubated at 37°C for 14 h, and the absorbance at 600 nm was detected with the microplate reader each hour throughout the incubation. Non-treated bacteria were served as a negative control. All sets were conducted in triplicate.

#### Time-Killing Assays of GHc and GHd

The kinetics of bacterial killing assay was measured as previously described (Abraham et al., 2014). *S. mutans* in the logarithmic phase was diluted in BHI to obtain a concentration of 2 × 10^6^ CFU/ml. GHc or GHd was prepared in PBS in a two-fold serial dilution. Then, 200 μl of each peptide at different concentrations (final concentration of 0.5×, 1× and 2× MIC) and the same volume of diluted bacterial suspension were mixed individually. The mixtures were incubated for 0, 30, 60, 90, 120, and 180 min at 37°C under anaerobic conditions. PBS without peptides was used as a negative control. Aliquots of 20 μl were plated on BHI agar plates after a 100-fold dilution in BHI at different times. The bacterial colonies were counted after incubation for 18 h at 37°C. The results were determined and presented as the mean of three repeats.

#### Stability Analysis of GHc and GHd

To evaluate the stability of GHc and GHd, both peptides were tested against various degradating or inactivating factors, including temperature, NaCl, pH or ultraviolet (UV) irradiation. The antimicrobial activities of GHc and GHd were evaluated after they were pretreated at 40–100°C for 30 min. For the NaCl stability assay, GHc and GHd (final concentration of 2 mM) were incubated with different concentrations of NaCl solution (final concentrations of 0.15, 0.2, 0.3, 0.4 M) at 25°C for 3 h. The NaCl solutions with different concentrations served as negative controls. GHc and GHd were dissolved in solutions with pH values ranging from 2 to 10 and incubated at 25°C for 3 h. The solutions with different pH values served as negative controls. For measurements of UV irradiation sensitivity, GHc and GHd were exposed to UV irradiation from 10 to 30 min. *S. mutans* in the exponential phase was mixed with warm BHI agar media to prepare bacteria-containing agar plates (final bacterial density of 1 × 10^8^ CFU/ml). Wells with 6 μm diameters were punched on the plates, and 10 μl of pretreated GHc or GHd (2 mM) were added to each well. The same amount of untreated peptides and sterile water were used as positive and negative controls. After incubation at 37°C for 16 h, the diameters of the inhibition zones were measured.

### Antibiofilm Activities of GHc or GHd Against *Streptococcus mutans* Biofilm

#### Inhibition of Bacterial Attachment

The effect of GHc or GHd on biofilm formation of *S. mutans* was evaluated as previously described with minor modification (Shang et al., 2014; Yuan et al., 2019). Bacteria were grown to logarithmic phase in BHI at 37°C and diluted in BHI (containing 3% sucrose) to obtain a concentration of 2 × 10^6^ CFU/ml. *S. mutans* suspensions were seeded into 96-well microplates (Corning, USA) in the presence of different concentrations of each peptide (final concentrations of 0.5×, 1×, and 2× MIC). PBS (pH 7.2) was served as a negative control. The plates were incubated at 37°C for 24 h. The biomass and metabolic activity of *S. mutans* biofilm were determined with the crystal violet (CV) assay and methyl thiazol tetrazolium (MTT) assay, as described below. Minimum biofilm inhibition concentration (MBIC_50_) was defined as the lowest concentration of peptides at which the biofilm formation was inhibited by at least 50%.

#### Biofilm Disruption Assay

The effect of GHc or GHd on preformed biofilm of *S. mutans* was evaluated as previously reported (Shang et al., 2014; Yuan et al., 2019). *S. mutans* was grown to logarithmic phase in BHI at 37°C and diluted in BHI (containing 3% sucrose) to 1 × 10^6^ CFU/ml. Two hundred microliters of *S. mutans* were seeded in each well of 96-well plates in anaerobic conditions at 37°C for 12 or 24 h. Then, the supernatants were gently removed, and 200 μl of the peptides diluted with media (final concentrations of 0.5×, 1×, and 2× MIC) were added to each well. Sterile PBS (pH 7.2) was used as a negative control. The plates were incubated at 37°C for 24 h. The biomass and metabolic activity were determined with the CV and MTT assay as described below. Minimum biofilm reduction concentration (MBRC_50_) was defined as the lowest concentration of peptides required to eradicate the preformed biofilm by at least 50%.

#### Evaluation of Biofilm With Crystal Violet or Methyl Thiazol Tetrazolium

Biofilm metabolic activity and biomass were determined using CV and MTT assays (Mishra and Wang, 2017). After incubation, the cultures were gently discarded, and the biofilms were rinsed three times with PBS (pH 7.2) to remove unattached bacteria. Then, the biofilms were fixed with 200 μl of anhydrous methanol for 15 min and stained with 0.5% CV for 15 min at 25°C. The excess CV was washed away with PBS, and 200 μl of methanol anhydrous were added to dissolve CV stained on biofilms. The biofilm biomass was determined by measuring the absorbance of CV at OD_560_ using a microtiter plate spectrophotometer.

For the MTT assay, the cultures were removed, and the plates were air-dried after incubation for initial cell attachment or biofilm formation assay. Five microliters of MTT (5 mg/ml) and 100 μl PBS were added to each well and incubated for 3 h at 37°C. Then, the supernatants were removed, and 150 μl of dimethyl sulfoxide (DMSO) was added. The absorbance of the purple product at 560 nm was measured using a microtiter plate spectrophotometer.

### The Antibacterial Mechanism of GHc and GHd Against Planktonic *Streptococcus mutans*

#### Nucleic Acid Leakage *via* the Bacterial Membrane

A nucleic acid leakage assay was carried out to detect the integrity of the bacterial cell membrane (Gazoni et al., 2018). *S. mutans* in the exponential phase was diluted to 2 × 10^8^ CFU/ml in 0.9% NaCl and treated with GHc or GHd at 0.5 × MIC, 1 × MIC, or 2 × MIC at 37°C for 0, 30, 60, 90, 120, and 150 min. After incubation, *S. mutans* was collected by centrifugation at 8,000 rpm for 10 min at 4°C. The amount of nucleic acid leakage through the bacterial membrane was determined by measuring the absorbance at 260 nm. Saline was used as a negative control. Each experiment was conducted three times. During the leakage assay, the viability of bacteria was monitored by plating method described in Section “The Antibacterial Mechanism of GHc and GHd Against Planktonic *Streptococcus mutans*.”

#### Bacterial Membrane Integrity Assay

The integrity of the bacterial cell membrane was determined by a live/dead BacLight bacterial viability assay (Shagaghi et al., 2016). Considering the cell washing process might lead to the loss of bacteria, the high concentration of bacterial inoculation was used. *S. mutans* in the exponential phase was diluted to 2 × 10^8^ CFU/ml in 0.9% NaCl. Five hundred microliters of the bacterial suspension were treated with the equal volume of GHc or GHd (final concentrations of 0.5×, 1× or 2× MIC) at 25°C for 60 min. Isopropyl alcohol and sterile 0.9% NaCl served as the positive and negative controls. *S. mutans* was collected and incubated with the LIVE/DEAD^®^ BacLight^™^ Bacterial Viability Kit in the dark for 15 min. After incubation, excess fluorescence was gently removed, and the bacteria were observed by confocal laser scanning microscopy with a 63× oil immersion objective (CLSM, Leica, TCS-Sp8). The cell viability was calculated by BioFilmAnalyzer v.1.0 software (Bogachev et al., 2018).

#### Morphological Observation by Scanning Electron Microscopy

*S. mutans* (1 × 10^8^ CFU/ml) was cultured in the presence of GHc or GHd (1× MIC, 5× MIC and 20× MIC). Saline was used as a negative control. After incubation for 60 min at 37°C, *S. mutans* was harvested and washed three times with saline. *S. mutans* was then fixed in 2.5% glutaraldehyde for 3 h, followed by a wash with saline. After dehydration by ethanol solutions with serial concentrations of 30, 50, 70, 90, and 100% for 15 min, *S. mutans* was lyophilized overnight. The lyophilized samples were coated with gold. *S. mutans* was photographed by scanning electron microscopy (SEM, Phenom XL, Holland) at a magnification of 40,000× (operating voltage was 10 kV).

#### DNA Binding Experiments

DNA-binding activities of GHc or GHd were investigated by DNA gel retardation (Pan et al., 2018). In order to extract enough DNAs to identify on agarose gel electrophoresis, the high concentration of bacterial inoculation was used. *S. mutans* in the exponential phase was diluted to 1 × 10^9^ CFU/ml in 0.9% NaCl. Total DNA was extracted from *S. mutans* by using a bacterial DNA extraction kit (Sangon Biotech Co., Ltd., Shanghai, China). The concentration and purity of the extracted DNA were evaluated with an optical density ratio of 260–280 nm. The extracted DNA was dissolved in 50 μl of citrate-EDTA buffer (pH 9.0) at a final concentration of 3 mg/ml. Five microliters of the extracted DNA (3 mg/ml) were mixed with the same volume of different concentrations of GHc or GHd (final concentrations of 0.125, 0.25, 0.5, and 1 mM) at 37°C for 30 min. The mixture was evaluated by electrophoresis using a 0.8% agarose gel, and the migrated DNA was visualized by a Gel imaging system (Bio-Rad, Hercules, USA).

### The Antibiofilm Mechanism of GHc and GHd Against *Streptococcus mutans*

#### Biofilm Observation by Light Microscopy

*S. mutans* biofilms treated with GHc or GHd were observed by light microscopy as previously described with minor modifications (Packiavathy et al., 2014). *S. mutans* was cultured to exponential phase and adjusted to a final density of 2 × 10^6^ CFU/ml with BHI supplemented with 3% sucrose. Two hundred microliters of *S. mutans* were seeded in each well of a 24-well plate (Corning, USA) containing circular glass slides (diameter of 12 mm). In the biofilm attachment assay, 200 μl of GHc or GHd (final concentrations of 0.5×, 1× and 2× MIC) was added to each well and cultured at 37°C for 24 h. For the biofilm disruption assay, 200 μl of *S. mutans* were seeded in each well of a 24-well plate containing glass slides and incubated for 12 and 24 h individually. The cultures were gently removed, and the biofilms were washed with PBS. Sequentially, 200 μl of GHc or GHd diluted in media (final concentrations of 0.5×, 1×, and 2× MIC) were added, with PBS (pH 7.2) serving as the negative control. After culturing for 24 h, the plates were washed with PBS (pH 7.2) and stained using 0.5% CV for 15 min. The biofilms were observed under a light microscope (Motic BA410, China).

#### Extraction of Extracellular Polysaccharides

The bacterial EPSs from *S. mutans* were isolated as previously described, with slight modifications (Gulube and Patel, 2016). Since the peptides at the concentrations near the MIC might decrease the bacterial viability and interfere in the results, sub-MIC concentrations were used to ensure the bacterial growth was not affected by additioned peptides according to the growth curve assay in Section “Growth Curve Assay of GHc and GHd.” *S. mutans* bacteria in the exponential phase were diluted to a final density of 2 × 10^6^ CFU/ml with BHI supplemented with 1% sucrose. Five milliliters of *S. mutans* were added to the same volume of GHc or GHd at the final concentrations ranging from 0.25× MIC to 0.5× MIC and cultured at 37°C for 20 h. After incubation, the culture was harvested by centrifugation at 10,000 rpm for 20 min at 4°C. The precipitate was washed twice with water, and the supernatant containing water-soluble glucans was collected. All the precipitates obtained was washed with 10 ml of 0.1 M NaOH and centrifuged at 10,000 rpm for 20 min. The supernatants were mixed with 30 ml of chilled 95% ethanol and placed overnight at 4°C to precipitate out water-insoluble glucans. The phenol-sulfuric acid method was carried out to measure the quantification of EPSs. One hundred microliters of chilled 5% aqueous solution of phenol and the same volume of sulfuric acid were mixed with EPSs and placed at 25°C to react. The absorbance of the mixture was measured at 490 nm using a microtiter plate spectrophotometer.

#### RNA Extraction and RT-qPCR Assay

RT-qPCR was performed as previously described with slight modifications (Durso et al., 2014). *S. mutans* in the exponential phase was diluted to 2 × 10^7^ CFU/ml. Then, 500 μl of the bacterial suspension were added to the same volume of GHc or GHd (final concentrations of 0.25× and 0.5× MIC) and cultured at 37°C for 8 h. After incubation, *S. mutans* were collected, and total RNA was extracted by using Trizol™ reagent (Invitrogen, CA, USA). The RNA concentration was evaluated at 260 nm. One microgram of extracted RNA was used as a template to synthesize complementary DNA (cDNA) in a 20 μl final reaction volume using the All-in-One First-Strand synthesis Kit (NOVA, CA, China). The effect of GHc or GHd on the expression of *gtf* genes (*gtf*B, *gtf*C, and *gtf*D) was evaluated by real-time quantitative PCR (qRT-PCR) conducted on the Applied Biosystems ABI 7500 (Applied Biosystems^™^). The primers were listed in Table 1. The reaction mixtures (25 μl) consisted of 2× RealUniversal PreMix with SYBR Green I (TIANGEN, Beijing), template cDNA and primers (10 mM). Thermal cycling conditions were conducted according to the manufacturer’s instructions. A 40-cycle thermal reaction was programmed, consisting of denaturation for 15 min at 95°C, annealing for 20 s at 58°C, and extension for 25 s at 72°C. Recombinase A (*rec*A) was served as the reference gene. The level of *gtf* expression was normalized in accordance with the 2^−△△CT^ analysis method (Kim et al., 2016). All sets were tested in triplicate.

**Table 1:** Primers used for RT-qPCR.

Genes	Primer sequences	Primer length
*rec*A-F	GTGCGGAGATTGACGGAGATA	21
*rec*A-R	CTTCCTTAAATGGTGGAGCAAC	22
*gtf* B-F	TGCCGCAGTCCCTTCTTATTC	21
*gtf* B-R	GCCATGTATTGCCCGTCATCT	21
*gtf* C-F	GTGCGCTACACCAATGACAGAG	22
*gtf* C-R	GCCTACTGGAACCCAAACACCTA	23
*gtf* D-F	TACCTTGGGCACCACAACACT	21
*gtf* D-R	TGCCGCCTTATCATCCTCACT	21

### The Toxicity Assay of GHc or GHd

#### Hemolytic Assays of GHc or GHd on Human Red Blood Cells

Human red blood cells (hRBCs) were washed with PBS (pH 7.2) until the supernatant was colorless and clear. The cells were resuspended in PBS (pH 7.2) at 2 × 10^8^ cells/ml. Two hundred microliters of hRBCs and the same volume of serially diluted peptides (final concentrations of 50, 75, 100, 150, and 200 μM) were mixed and incubated at 37°C for 60 min. After incubation, the mixture was centrifuged at 3,500 rpm for 10 min to collect the supernatant. The supernatant (150 μl) was removed to a new 96-well plate, and the absorbance was detected at 450 nm. An equal volume of PBS was used as a negative control, and 0.1% Triton X-100 was used as a positive control. The hemolysis of GHc and GHd were evaluated in the presence of *S. mutans* (1 × 10^6^ CFU/ml) (Mishra et al., 2018). The 50% of hemolysis (HC_50_) value was calculated.

#### Cytotoxicity of GHc or GHd on Human Oral Cells

Human oral epithelial cells (HOECs) were purchased from Shrdio Company (Nanjing, China). The cytotoxicity of GHc or GHd on HOECs was evaluated by a Cell Counting Kit-8 (CCK-8) assay (Beyotime Biotechnology, China) (Alaufi et al., 2017; Liu et al., 2017). HOECs were grown in Dulbecco’s modified Eagle’s medium (DMEM) containing 10% fatal bovine serum (FBS) and cultured in a humidified atmosphere of 5% CO_2_ at 37°C. Before the assay, 100 μl of cells (1 × 10^5^ cells/ml) were seeded in each well of 96-well plates and cultured at 37°C for 24 h. After the media was removed, 200 μl GHc or GHd with various concentrations (6.3, 12.5, 25, 50, 100, and 200 μM) in DMEM were added and incubated for 60 min. After treatment, the peptide solution was decanted, and 200 μl DMEM containing 10% FBS were added and incubated for 24 h. Thereafter, 10 μl of CCK-8 solution were added to each well, and the mixture was incubated for 3 h. The absorbance was measured at 450 nm by a microplate reader. The negative control was treated with PBS only. Cisplatin (100 μM) was used as the positive control. Three independent experiments were performed. The cell viability was calculated by the following formula: Percentage of viability = [(A_450 sample_ − A_450 blank_)/(A_450 negative control_ − A_450 blank_)] × 100%. Before this assay, the MICs of GHc or GHd in DMEM were evaluated. The media showed negligible effect on the antibacterial activity.

### Statistical Analysis

All experiments were conducted at least in triplicates at three separate assays. GraphPad Prism 6 for Microsoft Windows was used for the statistical analysis. Differences between the treated and untreated groups were analyzed by Student’s *t* test. The data were presented as mean ± standard deviations. The data were considered statistically significant when ^*^p < 0.05 or ^**^p < 0.01.

## Results

### Circular Dichroism Assays and Predicted Peptide Structure

**Figure 1:**
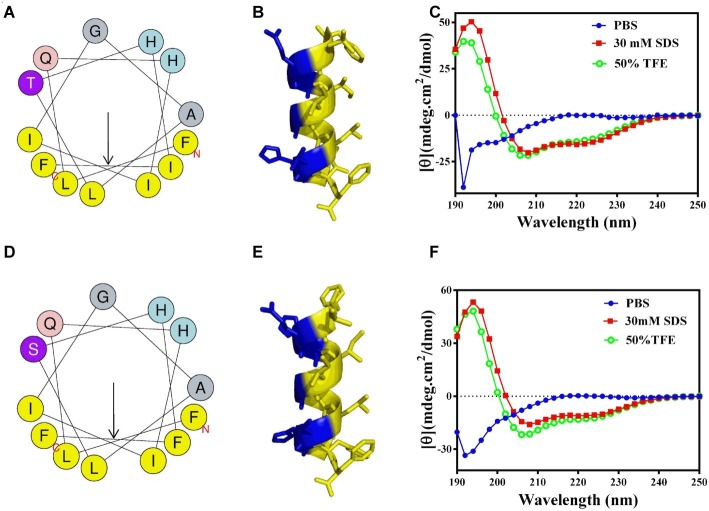
The helical wheel projections, 3D structures, and CD analysis of **(A–C)** GHc and **(D–F)** GHd. **(A,D)** The helical wheel projections are schematized on the left side. The arrows represent the direction of summed vectors of hydrophobicity. **(B,E)** 3D structures were calculated with PyMOL. CD spectra of **(C)** GHc and **(F)** GHd in PBS, 50% TFE or 30 mM SDS were analyzed. The final concentrations of GHc and GHd were 150 μM. Data are expressed as the mean residue ellipticities.

The 2D structures of GHc and GHd were determined by CD spectroscopy in PBS (aqueous solution), 50% TFE or 30 mM SDS (membrane mimetic environment). In PBS, both GHc and GHd revealed a single peak of characteristic absorption at 200 nm, which was indicative of predominantly random coil structures in aqueous solution. In contrast, GHc or GHd exhibited two negative peaks at approximately 208 and 222 nm and one positive peak at approximately 192 nm in a membrane mimetic environment, demonstrating that GHc and GHd were predominantly α-helical structures. The helical wheel diagram and 3D structures showed that all the hydrophobic amino acid residues of GHc or GHd were located on one side, whereas the hydrophilic amino acid residues were on the other side of the helix, leading to the formation of a hydrophobic face (Figure 1).

### Antibacterial and Antibiofilm Activity of GHc and GHd

#### Antibacterial Activity and Killing Kinetics of GHc and GHd

**Figure 2:**
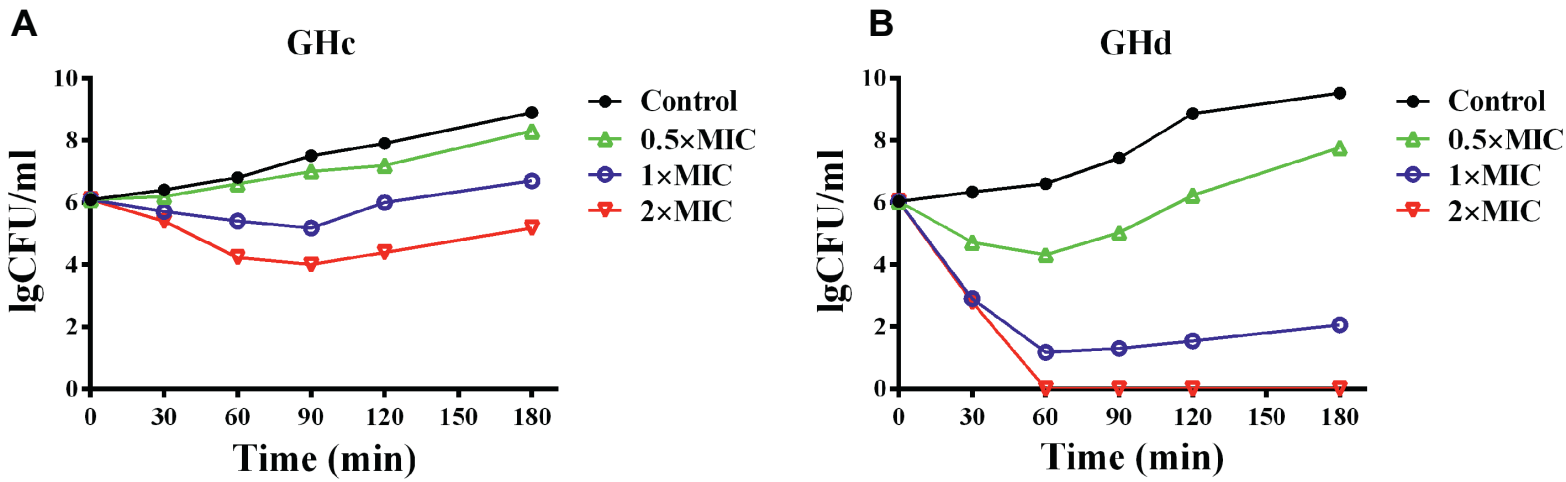
The time-killing kinetics of **(A)** GHc and **(B)** GHd against *S. mutans*. The bacteria were treated by GHc or GHd at the concentration of 0.5×, 1×, and 2× MIC. The bacteria untreated with the peptides were served as control. The experiments were performed at least in triplicate. The data were presented as mean ± SD.

GHc and GHd exhibited bactericidal activity against *S. mutans* with MICs of 12.6 and 13.1 μM, respectively (Supplementary Figure S3). The measured MBC of GHc was higher than 50 μM, while that of GHd was only 26 μM (Table 2), indicating that GHd had better bactericidal effects against *S. mutans*. Time-killing curves of GHc and GHd were carried out with the peptides at concentrations of 0.5×, 1×, and 2× MIC (Figure 2). Compared to the negative control, which showed a fast increase in bacterial colonies, GHd showed a concentration-dependent and time-dependent bactericidal effect on *S. mutans*, taking 60 min to kill all bacteria at the concentration of 2× MIC. GHc showed less bactericidal potency toward *S. mutans*.

**Table 2:** The antibacterial and antibiofilm activities of GHc and GHd against *S. mutans.*

Peptides	MIC (μM)	MBC (μM)	MBIC_50_ (μM)	MBEC_50_ (μM)^*^	
				12 h	24 h
Temporin-GHc	12.6	>50	6.3	25	>50
Temporin-GHd	13.1	26	6.6	26	>50

* *MBEC_50_ was determined with the biofilms preformed for 12 and 24 h*.

#### Stability Analysis of GHc and GHd

The stability of GHc or GHd was shown in Supplementary Figure S4. GHc and GHd retained antibacterial activity against *S. mutans* after pretreatment at various temperatures, which was not significantly different from the negative control (*p* > 0.05). The groups treated with various NaCl concentrations indicated no significant differences from the untreated control groups (*p* > 0.05). GHc and GHd also demonstrated stability in the presence of low or high pH and UV irradiation. These results suggested that the heating temperature, NaCl concentration, pH, and UV irradiation could not change the antibacterial activity of GHc and GHd against *S. mutans*.

#### Antibiofilm Activity of GHc and GHd

CV and MTT assays revealed that the inhibition rates of GHc and GHd on the initial attachment of biofilm formation at 0.5× MIC were 50–60% (Supplementary Figure S5). The MBIC_50_ of GHc and GHd were 6.3 and 6.6 μM, respectively (Table 2). After *S. mutans* biofilms were performed for 12 h, the reduction rates of the two peptides on *S. mutans* biofilm at 1× MIC decreased to approximately 38% and about 60–65% at 2× MIC (data not shown). Thus, the MBRC_50_ were 25 and 26 μM. After the *S. mutans* biofilms had grown for 24 h, the destruction rates of GHc and GHd at 1× MIC were approximately 10–18%.

### The Antibacterial Mechanism of GHc and GHd Against Planktonic *Streptococcus mutans*

#### Nucleic Acid Leakage Through Bacterial Membranes

To investigate whether GHc and GHd target to the bacterial membrane, the nucleic acid leakage assay in the cell culture media was tested. Generally, a macromolecular substance cannot penetrate the integral cell membrane. However, once the integrity of bacterial membrane is damaged, the macromolecules leak to extracellular environment and can be monitored. When *S. mutans* cells were treated with increasing concentrations of GHc or GHd, the absorption at 260 nm increased gradually, indicating that the membrane damage level and membrane permeability were enhanced, thus leading to continuous leakage of DNA (Supplementary Figure S6). When *S. mutans* was exposed to GHc for 150 min, the leakage of macromolecular substances in the bacteria increased gently. For GHd, it took 120 min to reach maximal leakage. This observation might relate to the efficient antimicrobial activity of GHd on *S. mutans*. Comparatively, the absorbance of the negative control groups slightly changed with increasing incubation time, which are the metabolites produced by the bacteria for life activities. During the DNA leakage assay, the viability of bacteria was measured. Even the DNA leakage increased obviously with the high concentration of GHc, GHc showed less bactericidal activity. However, the viability of bacteria treated by GHd declined sharply. This result was consistent with the time-killing curves.

#### Effect on Bacterial Membrane Integrity

**Figure 3:**
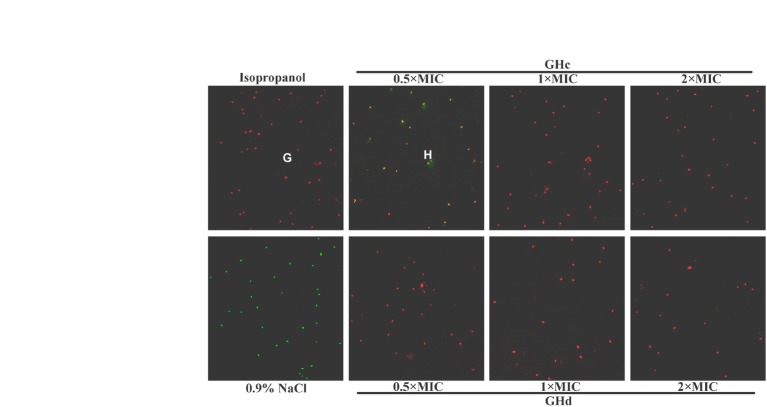
The effect of GHc and GHd on the membrane integrity of *S. mutans*. **(A)** Isopropanol was used as the positive control (PC), and **(E)** 0.9% NaCl was used as the negative control. The bacteria were treated with **(B–D)** GHc or **(F–H)** GHd at the concentrations of 0.5×, 1×, or 2× MIC for 60 min.

The effect of GHc and GHd on the membrane integrity of *S. mutans* was further determined with the LIVE/DEAD BacLight Bacterial Viability Kit (Figure 3). Compared with the positive control and negative control, both GHc and GHd induced a change in the membrane integrity of *S. mutans*. When treated with 0.5× MIC GHc, the bacterial cells stained orange, indicating that the intact membrane of bacteria treated with GHc had begun to change. When the peptide concentration was increased to 1× MIC, the cells were all red, suggesting that more GHc was required to disrupt the integrity of cell membranes. When treated with GHd at concentrations of 0.5×, 1×, and 2× MIC, the cells were all red, suggesting that the membrane permeability of *S. mutans* was significantly disrupted. The cell viability was calculated by BioFilmAnalyzer (Supplementary Figure S7). When *S. mutans* were exposed to the two peptides at the concentrations higher than 1× MIC, nearly all bacteria were killed.

#### Scanning Electron Microscopy Analysis

**Figure 4:**
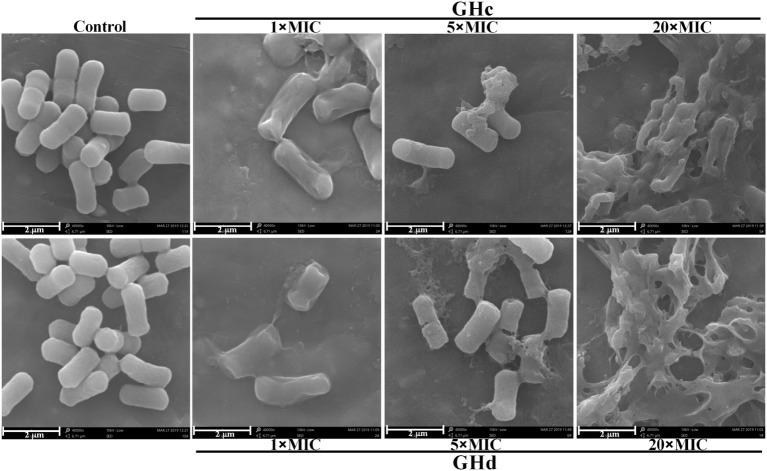
The morphological changes of *S. mutans* after treatment with GHc or GHd were observed by using the scanning electron microscopy. The bacteria were treated with 0.9% NaCl served as the negative control. The bacteria were treated with the peptides at the concentration of 1×, 5×, or 20× MIC for 60 min.

SEM was used to observe bacterial morphology changes after treatment with GHc or GHd (Figure 4). *S. mutans* cells treated with 0.9% NaCl were full and round with clear and smooth surfaces. After the bacteria were exposed to GHc or GHd for 60 min at 5× MIC, visible damage was observed. Most cells lost the intact and smooth membranes, leading to the leakage of intracellular materials. When the peptide concentration was increased to 20× MIC, all cells shriveled and became deflated, and the membranes were severely disrupted and adhered to each other.

#### Peptides Binding With DNA of *Streptococcus mutans*

Gel retardation experiments indicated that binding of GHc or GHd with *S. mutans* DNA *in vitro* was in a peptide concentration-dependent manner (Supplementary Figure S8). When the concentrations of GHc and GHd reached 0.5 mM, the DNA was completely retained in the wells and could not migrate into the gel.

### The Antibiofilm Mechanism of GHc and GHd Against *Streptococcus mutans*

#### Light Microscopy Image Analysis of *Streptococcus mutans* Biofilm

**Figure 5:**
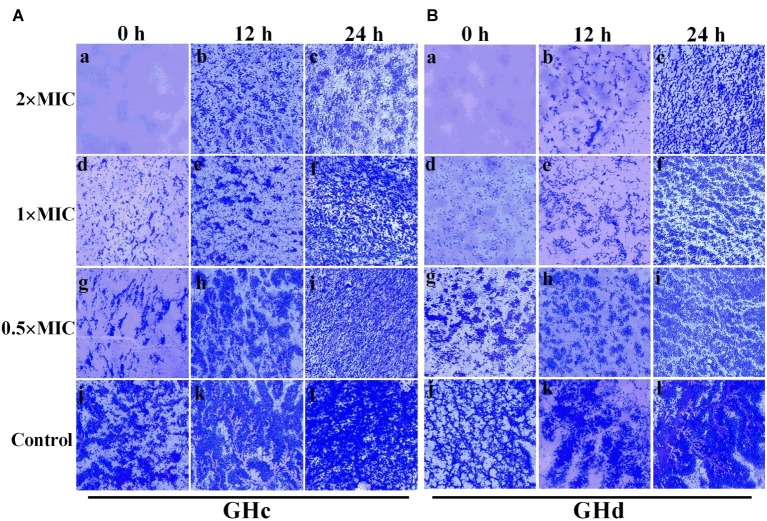
Effects of **(A)** GHc and **(B)** GHd on *S. mutans* biofilm observed by using light microscopy. **(a,d,g,i)** The planktonic bacteria were incubated with the peptides to detect the effect on the initial cell attachment of biofilm formation. For the analysis of the effect on the mature biofilms, the biofilms were performed for 12 or 24 h before exposed to the peptides. **(j,l)** The control *S. mutans* biofilms were treated with PBS. The biofilms were treated with GHc or GHd at **(a–c)** 2× MIC, **(d–f)** 1× MIC, and **(g–i)** 0.5× MIC. The biofilms were stained in dark blue.

GHc and GHd showed obvious inhibition of attachment at the beginning of biofilm formation (Figure 5). When the peptide concentration was 2× MIC, the attachment of planktonic bacteria was completely inhibited. The two peptides can also disrupt a preformed biofilm. Compared with GHc, GHd showed more potent disruption of a 12-h-old biofilm at 2× MIC, and only scattered and small bacterial clusters of biofilm were observed. Although the 24-h-old biofilm possessed highly organized structures, GHc and GHd could still disrupt the biofilm, contributing to a reduction in biofilm thickness and disordered structures.

#### Extracellular Polysaccharide Production

**Figure 6:**
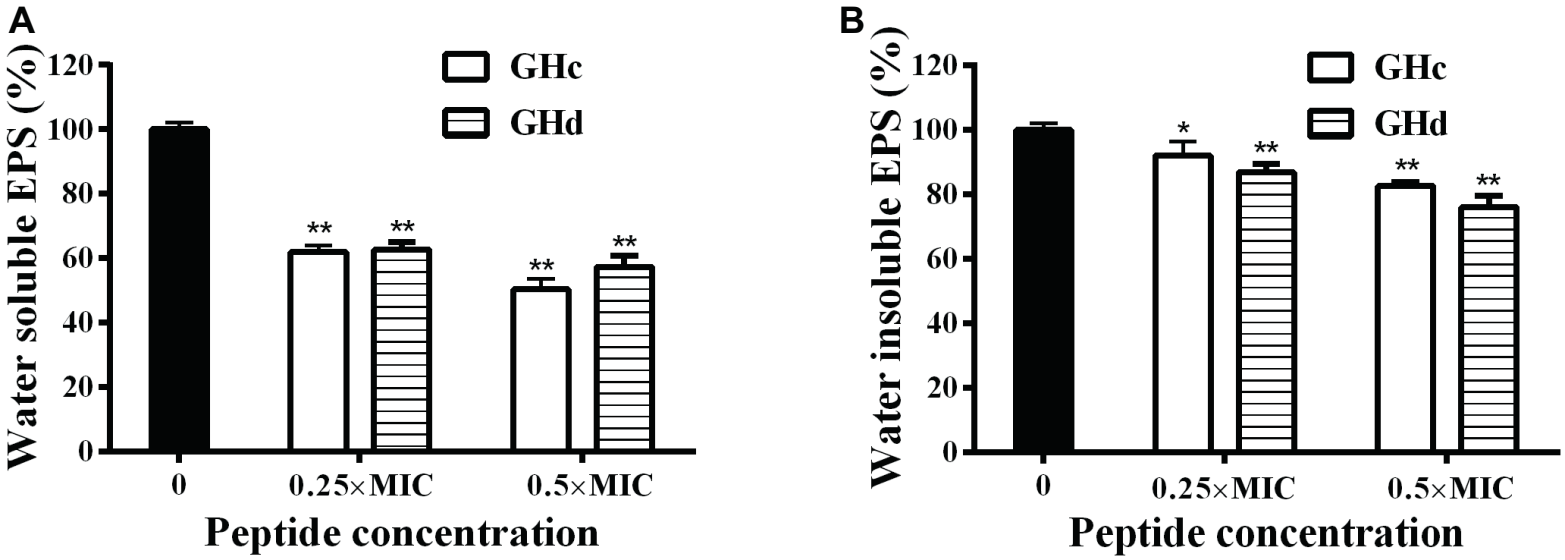
Effects of GHc and GHd on EPS produced by *S. mutans*. After the bacteria were treated with GHc or GHd at the concentration of 0.25× or 0.5× MIC, **(A)** the water-soluble EPS and **(B)** the water-insoluble EPS were measured. The untreated bacteria were served as control. ^*^p < 0.05, ^**^p < 0.01 indicate the significant difference.

Since that GHc and GHd at sub-MIC concentration did not influence the growth of bacteria (Supplementary Figure S9), the effect of GHc and GHd with the concentration of under sub-MIC on the EPSs produced by *S. mutans* was measured (Figure 6). The results revealed that there was a significant decrease in water-soluble and water-insoluble EPS production in the planktonic growth. The inhibitory rates of GHc and GHd on water-soluble EPS produced by *S. mutan*s at different concentrations (0.25× and 0.5× MIC) were 39–52 and 38–46%, respectively. The inhibition rates of water-insoluble EPS were 11–19 and 13.4–27.3%. GHd showed a higher level of inhibitory activity. These results were consistent with those observed in *S. mutans* biofilms assay by CV and MTT methods.

#### Effects of Peptides on *Streptococcus mutans* Gene Expression

**Figure 7:**
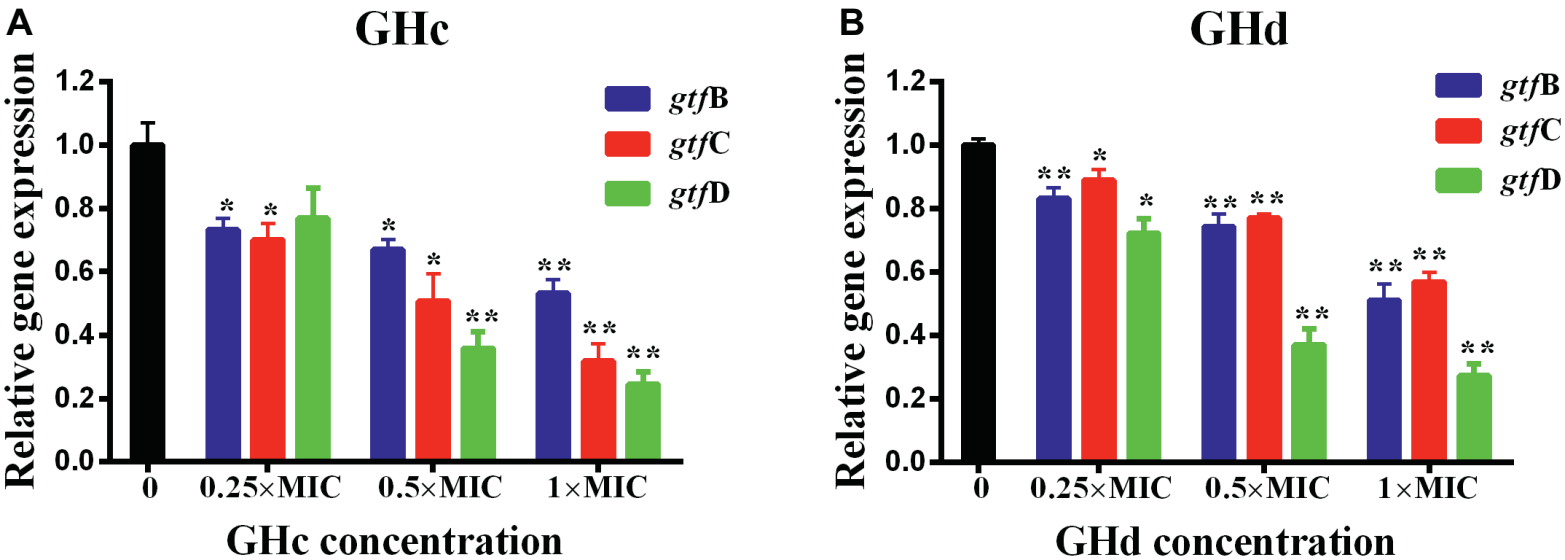
Effects of **(A)** GHc and **(B)** GHd on *gtfs* gene expression in *S. mutans* were quantified by quantitative real-time PCR, with recombinase A gene as an internal control. The experiments were performed at least three times. Data are shown as the mean ± SD. The asterisks (*) indicate the significant differences (^*^p < 0.05, ^**^p < 0.01).

The effect of GHc or GHd on the expression of genes related to *S. mutans* biofilm formation was shown in Figure 7. The *gtf* B and *gtf* C genes are responsible for the synthesis of water-insoluble polysaccharides, which are the main components of EPSs, while *gtf* D synthesizes water-soluble extracellular polysaccharides. Exposure of *S. mutans* to different concentrations of GHc or GHd caused a significant downregulation of *gtf* B, *gtf* C, and *gtf* D (*p* < 0.05). The relative expression levels of the *gtf* B, C, and D genes in bacteria treated with GHc were lower than 0.8 at concentrations of 0.25× and 0.5× MIC, indicating that the low concentrations of GHc downregulated the expression of *gtf*s, thereby affecting the synthesis of water-soluble and water-insoluble extracellular polysaccharides and interfering with or destroying the bacterial biofilm. Similarly, the relative expression levels of *gtf* B, C, and D genes in those treated with GHd were lower than 0.9, indicating that GHd also downregulated the expression of the biofilm-related genes.

### Cell Toxicity of GHc and GHd

#### Hemolytic Activity of GHc and GHd

The selectivity of GHc and GHd to *S. mutans* and erythrocytes were evaluated by measuring the hemolytic toxicity of antimicrobial peptides on human erythrocytes in the presence of bacteria. Compared with the treatment without *S. mutans*, GHc reduced its hemolytic toxicity at different concentrations of 12.5–200 μM in the presence of *S. mutans*, showing a 25% reduction in hemolytic toxicity at the concentration of 50 μM (Supplementary Figure S10). Similar results were obtained for GHd, with a 38% reduction at the concentration of 75 μM. The results showed that GHc and GHd were more selective for *S. mutans* and functioned to decrease toxicity toward hRBCs in the presence of *S. mutans*.

#### Cytotoxicity of Peptides to Oral Cells

**Figure 8:**
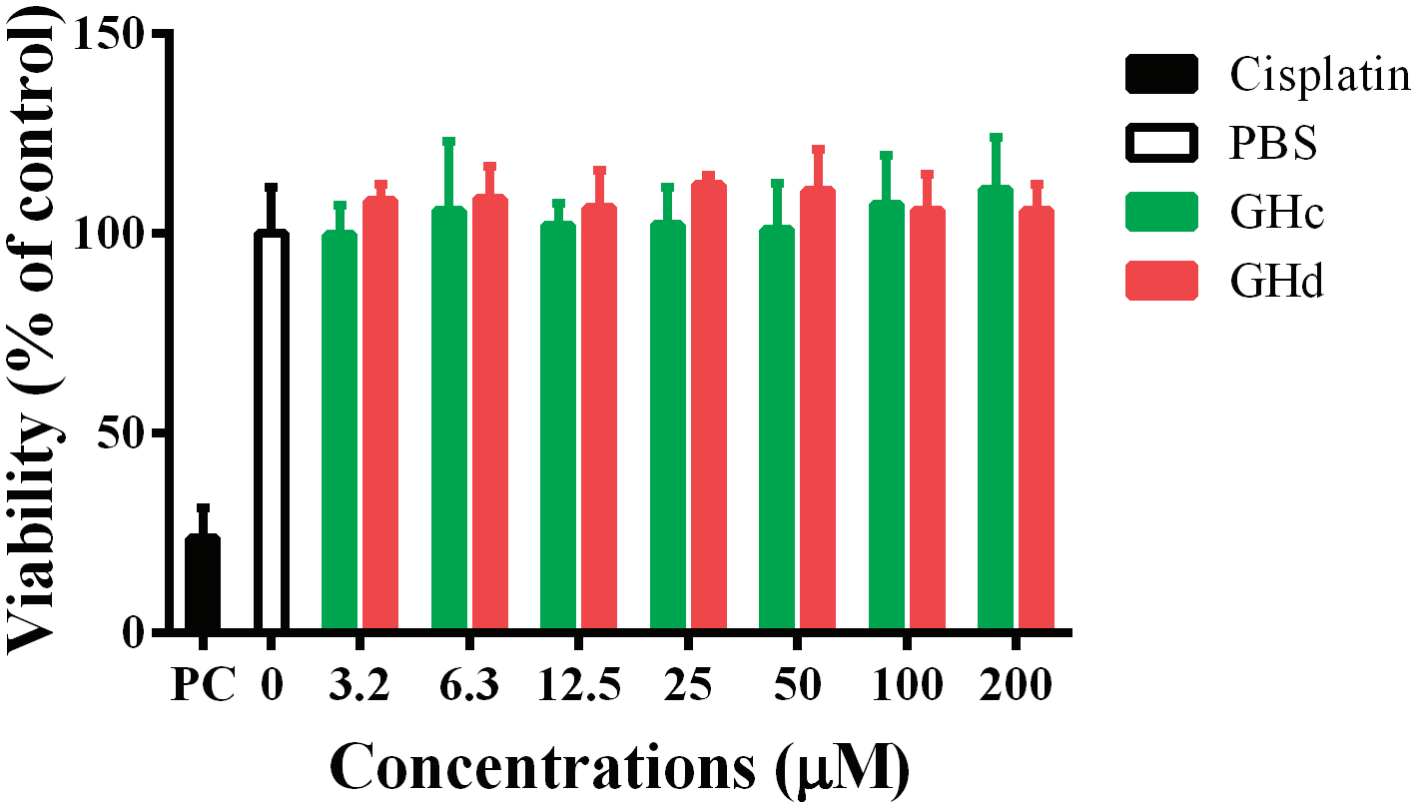
The cytotoxicity of **(A)** GHc and **(B)** GHd against HOECs were assayed. The cells were treated with the peptides at the concentration ranging from 3.2 to 200 μM for 60 min. PBS was served as the negative control. Cisplatin (100 μM) was used as the positive control. The experiments were performed at least in triplicate. Data are shown as the mean ± SD.

The cytotoxicity of GHc and GHd on HOECs was evaluated by the CCK-8 assay. The two peptides had no cytotoxicity on HOECs at concentrations up to 200 μM (Figure 8). On the other hand, GHc (at the high concentration of 100–200 μM) and GHd (3.2–200 μM) promoted cell proliferation in a dose-dependent manner.

## Discussion and Conclusion

The temporin AMPs, considered to be the shortest natural antimicrobial peptides, are various. They share the common characteristics of amidation at the C-terminal, but the primary structures and the number of positive charges vary (Michael, 2002; Rollinssmith et al., 2003; Abbassi et al., 2008). In our previous study, the cDNAs encoding GHc (FLQHIIGALTHIF) and GHd (FLQHIIGALSHFF) were cloned from the skin of *H. guentheri* on Hainan Island, China. Both showed a broad antimicrobial spectrum against Gram-positive bacteria, Gram-negative bacteria, and fungi (Dong et al., 2017). However, the temporin-GH derived from *H. guentheri* in Fujian, China only exhibited antimicrobial activity against the *S. aureus* with an MIC of 44.3 μg/ml (Zhou et al., 2006). A search in the APD3 database (Wang et al., 2015) showed that 10 out of 121 temporin AMPs have positively charged histidinyl residues and exhibited various antimicrobial spectra, indicating that histidine might not determine the AMPs’ preference for the microbial species. In comparison with the majority of temporin peptides that are charged with arginine or lysine, GHc and GHd are positively charged by two histidines located on one side of the predicted α-helical structure and have hydrophobic amino acid residues lining the opposite side, providing the common structure for α-helical AMPs. The results were consistent with the wheel projection model, in which GHc and GHd had a similar hydrophobic face consisting of F1, L2, I5, I6, L9, and F13 in common, but differing in position 10 and 12 with T and I for GHc and S and F for GHd, respectively. The two distinctive residues are both similar types of amino acids in the two peptides. Although GHc and GHd showed a similar MIC of 12.6 and 13.1 μM against *S. mutans*, the killing effect of GHd was much more powerful than that of GHc, suggesting that GHc might exert the bacteriostatic activity mainly and GHd showed the bactericidal action. There are many papers on AMPs showing the antimicrobial activity on *S. mutans* (Shang et al., 2014; Wang et al., 2018), but few reports about naturally occurring temporin members. Compared to Temporin-1CEb from *Rana chensinensis*, with an MIC of 50 μM against *S. mutans* (Shang et al., 2014), GHc and GHd demonstrated higher antimicrobial activities with lower MICs and stronger robustness against physical and chemical factors, including temperature, pH, NaCl, and ultraviolet irradiation.

GHc and GHd assumed random coil structures in aqueous solution, while existed as an α-helical structure in the membrane-mimicking environment. The change in 2D structure observed in different solvents and the membrane integrity assay indicated that GHc and GHd may combine with the bacterial membrane and affect the integrity of cell membrane. Therefore, we deduced that when GHc or GHd were close to the membrane, their structures changed from random coils to α-helices and bound to the membrane, leading to the increase of cell membrane permeability in *S. mutans* and leakage of the intracellular material. Meanwhile, the increased membrane permeability allows GHc and GHd to enter the cell and bind to DNA, contributing to a decrease in synthesis levels in bacterial DNA. These results are consistent with the findings of Li et al., who observed that the antimicrobial peptide APP damaged bacterial membrane permeability and bound to bacterial DNA to influence intracellular life activity (Li et al., 2016).

According to the literature, AMPs could be used to inhibit the formation of biofilms and to eradicate mature biofilms (Mishra and Wang, 2017; Verly et al., 2017). GHc and GHd significantly inhibited bacterial adhesion and growth of the *S. mutans* biofilm. Even after the biofilms of *S. mutans* were performed for 12 h, GHc or GHd could still destroy biofilms and reduce the density of bacterial biofilms at a concentration of 2× MIC. Bacterial EPSs are virulence factors necessary for *S. mutans* to adhere to the tooth surface and form cariogenic biofilms. EPSs are synthesized by glucosyltransferase (GTFs) and play crucial roles in supporting and forming biofilms (Flemming and Wingender, 2010; Gulube and Patel, 2016). GHc or GHd could inhibit the synthesis of water-soluble EPS and water-insoluble EPS in a dose-dependent manner. During the process of biofilm formation, GHc and GHd at a concentration less than Sub-MIC without showing antimicrobial activity were able to downregulate the expression of *gtf* B, *gtf* C, and *gtf* D. The inhibited expression of *gtf* genes in *S. mutans* biofilms might reduce the amount of glucosyltransferases in the biofilm, thereby reducing EPSs production; thus, biofilm formation or maturity can be inhibited by GHc and GHd. Both GHc and GHd showed a hemolytic activity on human blood cells, with the HC_50_ values of 95 and 50 μM, respectively; however, these values increased to more than 200 μM in the presence of *S. mutans*, indicating that GHc or GHd had a higher selectivity for *S. mutans*, which is beneficial in the treatment of infected cases. In addition, GHc and GHd were not cytotoxic to human oral epithelial cells.

In summary, the two peptides from *H. guentheri* GHc and GHd exerted antimicrobial activity against the oral pathogen *S. mutans* through increasing cell membrane permeability. They also impeded *S. mutans* biofilm attachment to teeth and disrupted its mature process; this impediment was mediated by downregulating the *gtfs* genes, which play a role in *S. mutans* biofilm formation. No cytotoxicity toward human oral epithelial cells and low cytotoxicity toward human red blood cells were observed for both studied peptides, rendering them promising candidates for further development of oral healthcare products.

## Data Availability Statement

The datasets generated for this study can be found in the Temporin-GHc (GenBank accession No: KU518308), temporin-GHd (GenBank accession No: KU518309).

## Ethics Statement

All experiments in this study were approved by the Ethics Committees of Hainan University.

## Author Contributions

YZ, YS, and MW designed and supervised the experiments. HZ performed the experiments, analyzed the data, and wrote the original manuscript. ZX, HW, and SZ performed parts of the experiments. YZ reviewed the manuscript. All the authors had read and approved the final manuscript.

### Conflict of Interest

The authors declare that the research was conducted in the absence of any commercial or financial relationships that could be construed as a potential conflict of interest.

## Footnotes

^1^http://heliquest.ipmc.cnrs.fr/
