# Supplementary material for: Antibacterial and Antibiofilm Activity of Temporin-GHc and Temporin-GHd Against Cariogenic Bacteria, Streptococcus mutans
Source: Front Microbiol. 2019 Dec 11;10:2854. doi: 10.3389/fmicb.2019.02854 (PMC6918509; doi:10.3389/fmicb.2019.02854)
Supplement: Supplementary file 1 [file Data_Sheet_1.pdf]

Supplementary Material

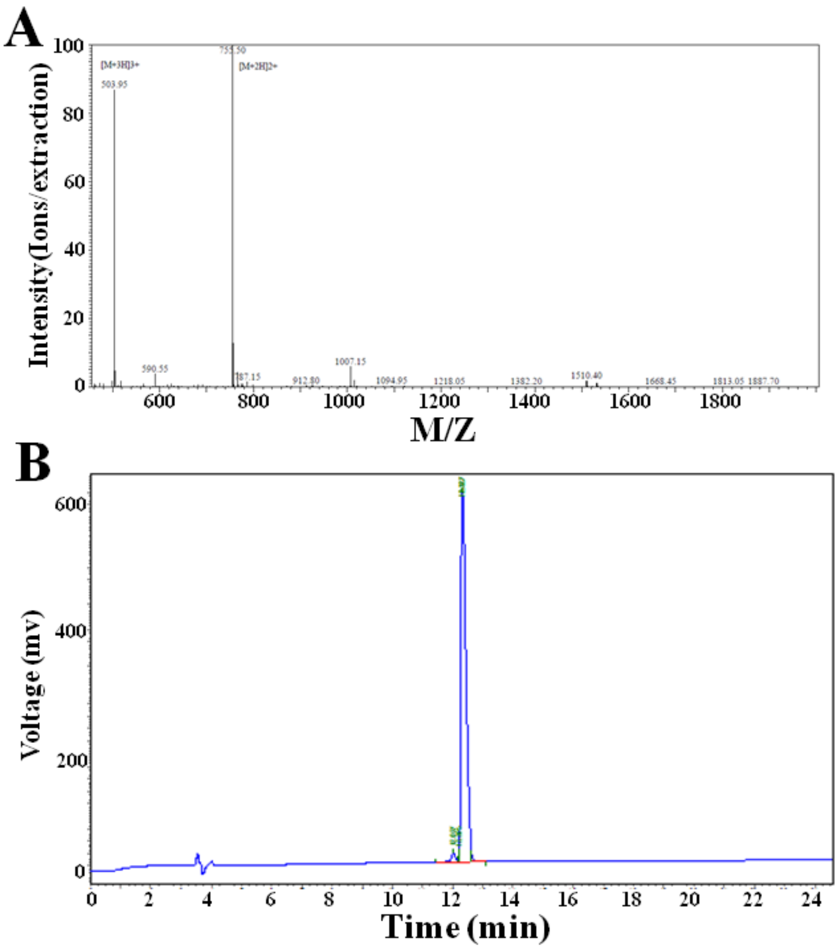

Supplementary Figure 1. (A) The mass spectrum and (B) RP-HPLC chromatogram of temporin-GHc.

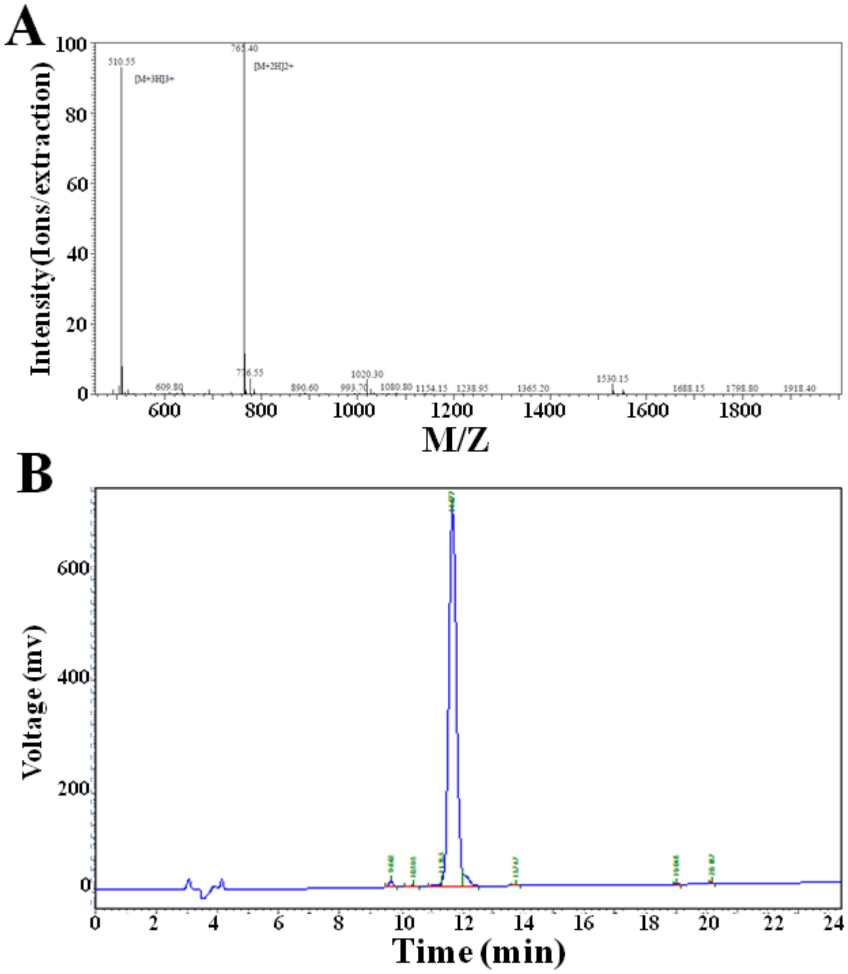

Supplementary Figure 2. (A) The mass spectrum and (B) RP-HPLC chromatogram of temporin-GHd.

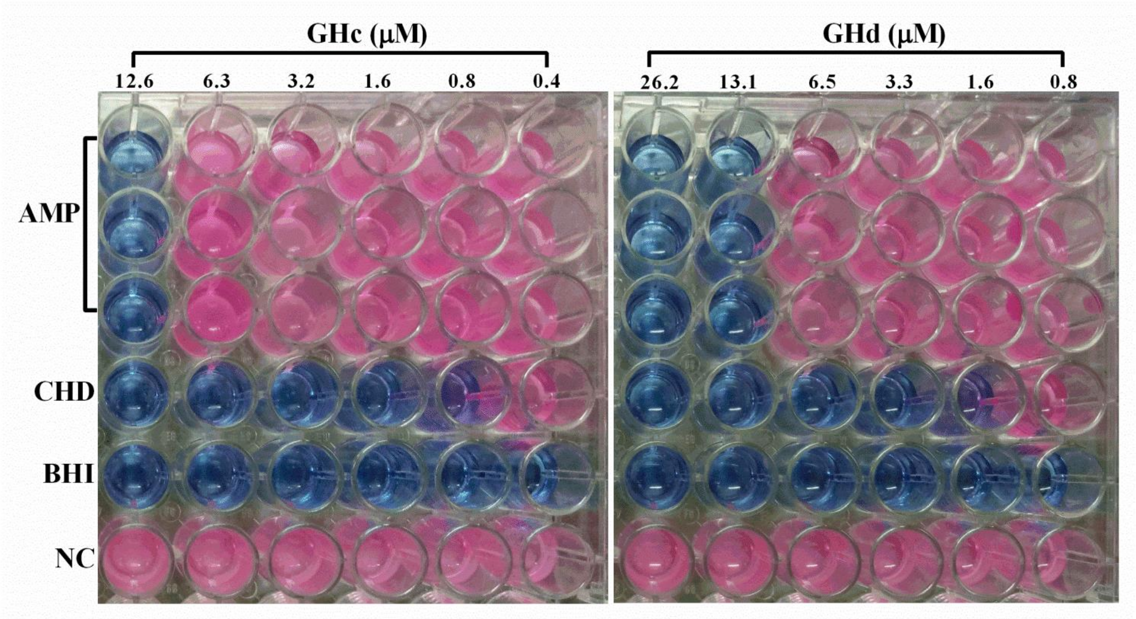

Supplementary Figure 3. Determination of MIC for GHc and GHd against *S. mutans*. After exposure to the peptides, the bacteria were incubated with resazurin sodium (final concentration of 14 μg/ml) for 2 h, and the lowest peptide concentration in blue was determined as the MIC. AMP indicated the bacteria were treated with GHc or GHd. CHD represented chlorhexidine with the concentrations ranging from 0.05-1.6 μM. BHI was brain heart infusion broth only. NC was negative control, in which the peptides were replaced by PBS.

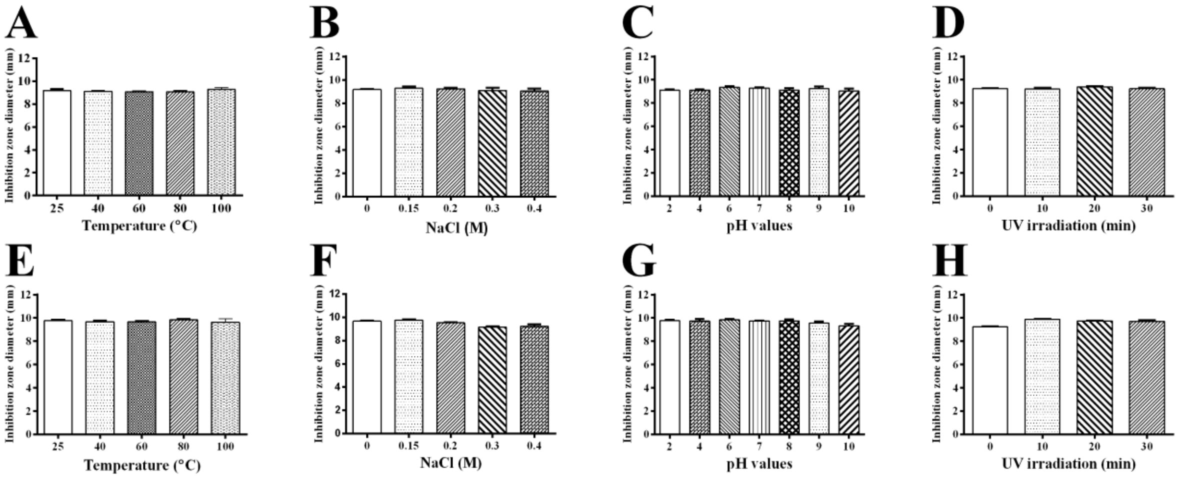

Supplementary Figure 4. The antimicrobial activity stability of (A-D) GHc and (E-H) GHd under virous conditions against *S. mutans* were measured. (A and E) After preheated in different temperatures, the antimicrobial activities of the peptides were assayed. The peptides pretreated at 25 ℃ were served as control. (B and F) The effect of NaCl with different concentration on the antimicrobial activities of the peptides was detected, with water used as control. (C and G) The antimicrobial activity was assayed at the range of pH 2 to 10. The treatment under pH 7 was functioned as control. (D and H) The UV irradiation effect was analyzed, with the untreated peptides as control. *$p < 0.05$ vs. control. The data with no * were not significantly different ($p > 0.05$).

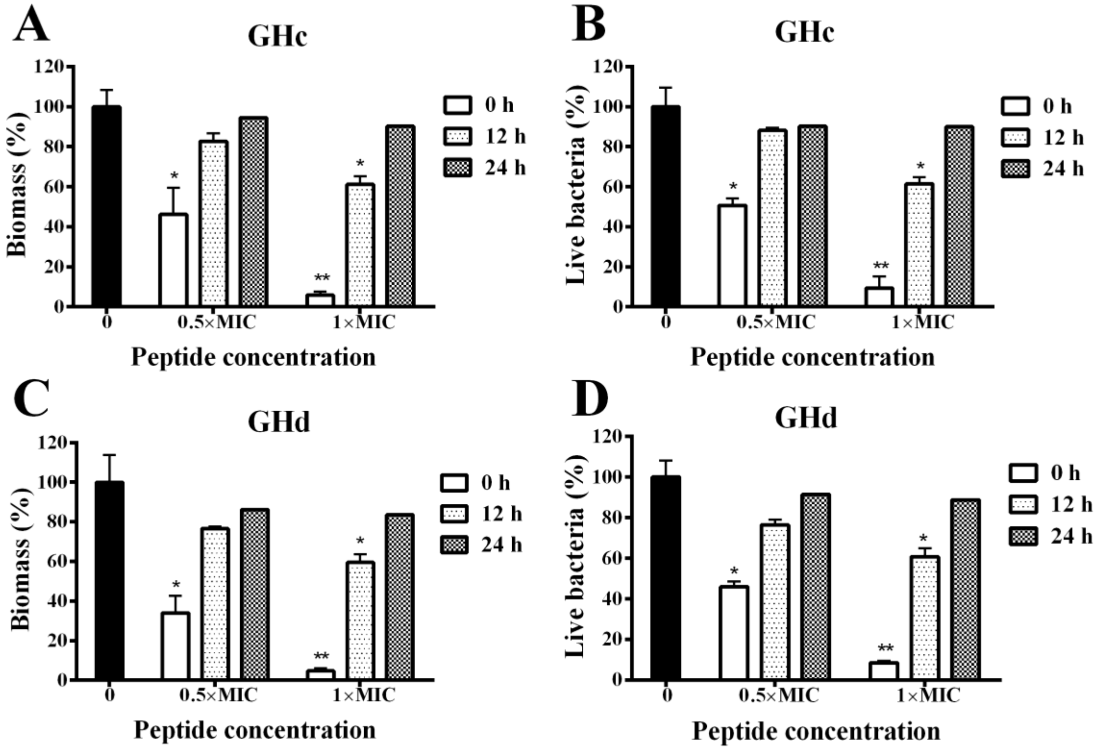

Supplementary Figure 5. Effects of (A, B) GHc and (C, D) GHd on *S.mutans* biofilm. The biofilm was stained with (A, C) CV and (B, D) MTT, respectively. Values represent the mean ± SD of three independent experiments. Before exposed to GHc or GHd, the biofilms were preformed for 0, 12, or 24 h.

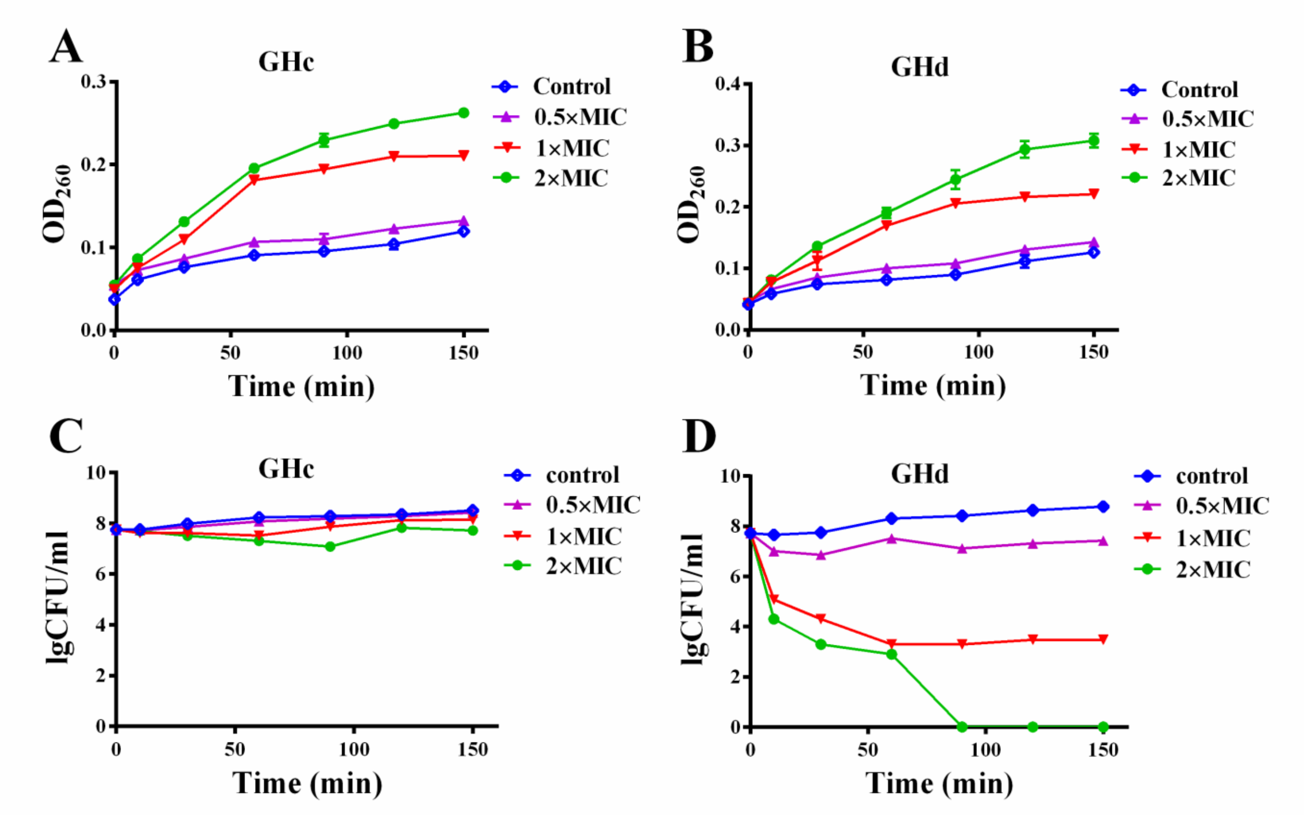

Supplementary Figure 6. The effect of (A) GHc or (B) GHd on the nucleic acid leakage was recorded at 260 nm, and (C and D) the viability of bacteria was monitored. *S. mutans* were treated by the peptides at the concentration of 0.5×, 1×, and 2×MIC, with 0.9% NaCl served as the negative control. Each value represents the mean ±SD of three independent experiments.

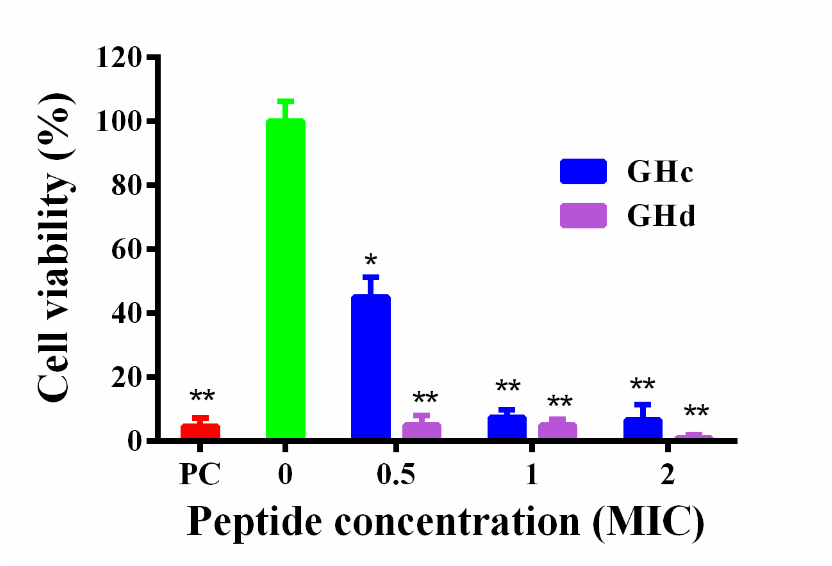

Supplementary Figure 7. The cell viability was calculated by BioFilmAnalyser on the assay of membrane integrity of *S. mutans* treated by GHc or GHd. Isopropanol was used as the positive control (PC), and 0.9% NaCl was used as the negative control. The bacteria were treated with or without GHc or GHd at the concentrations of 0.5×, 1× or 2×MIC for 60 min. * $p < 0.05$, ** $p < 0.01$, vs. the negative control were significant difference.

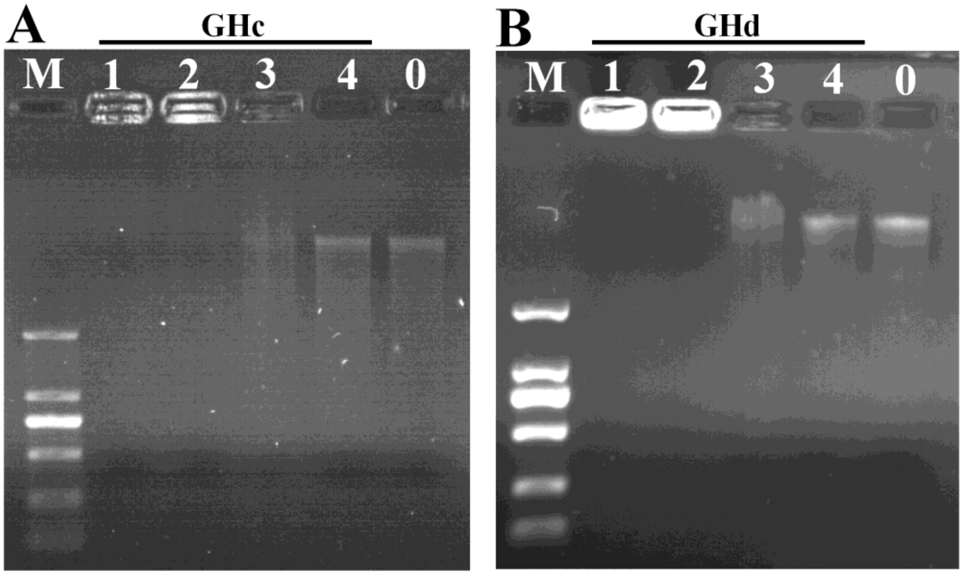

Supplementary Figure 8. *S. mutans* DNA binding analysis of (A) GHc and (B) GHd. Bands 1-4 contained the peptides at the concentrations of 1 mM, 0.5 mM, 0.25 and 0.125 mM, respectively. Band 0 was the genomic DNA treated with sterile water used as control, and M was the DNA marker.

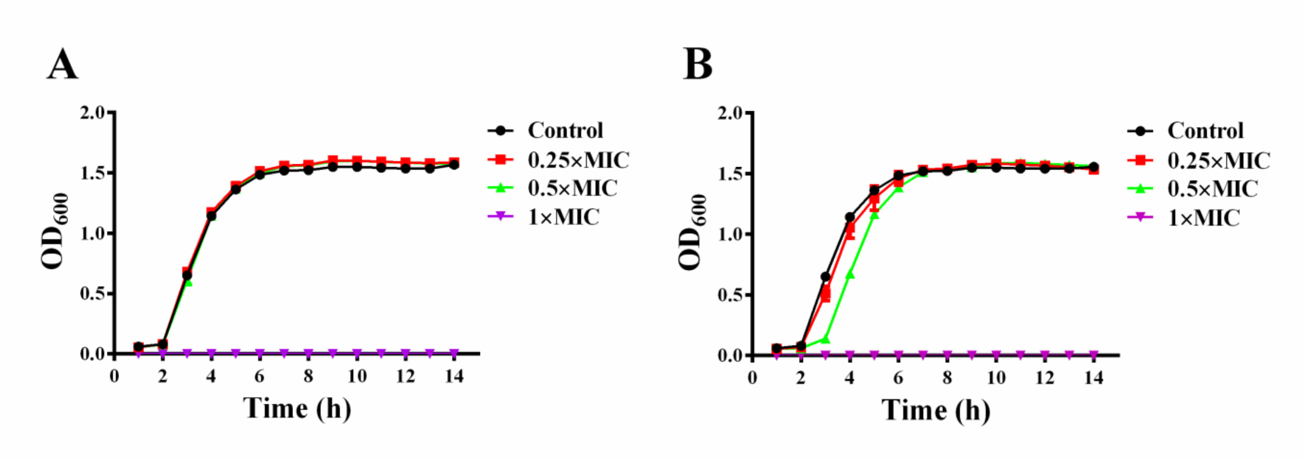

Supplementary Figure 9. (A) The growth curve of *S. mutans* treated by (A) GHc and (B) GHd at sub-MIC concentrations. The bacteria untreated with the peptides were served as control.

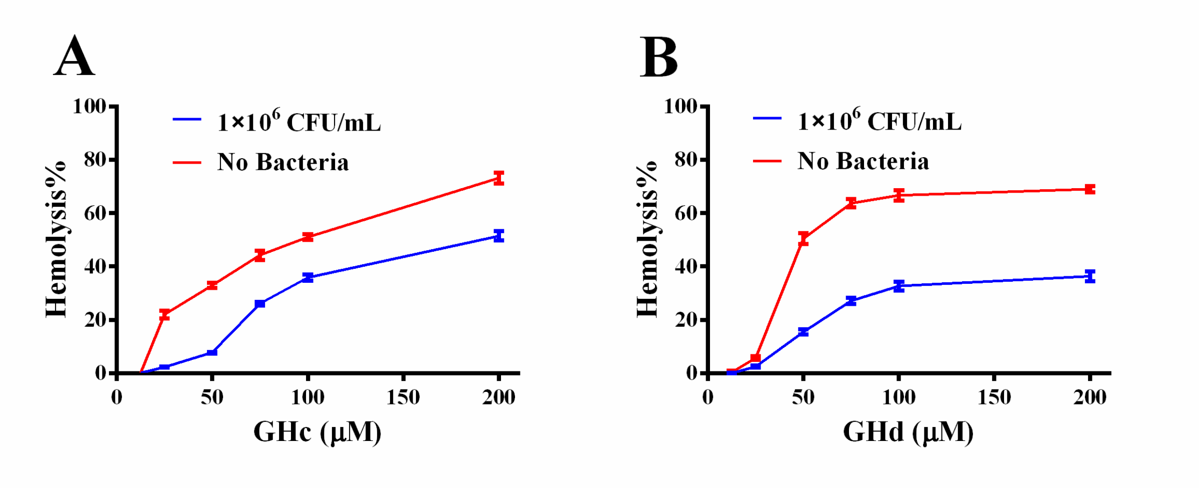

Supplementary Figure 10. Hemolytic activity of (A) GHc and (B) GHd were detected in the absence or presence of *S.mutans*. Human red blood cells were used. The experiments were performed at least in triplicate. Data are shown as the mean ± SD.
